# What is the optimal time point to assess patient-reported recovery after hip and knee replacement? a systematic review and analysis of routinely reported outcome data from the English patient-reported outcome measures programme

**DOI:** 10.1186/1477-7525-11-128

**Published:** 2013-07-30

**Authors:** John Patrick Browne, Hamad Bastaki, Jill Dawson

**Affiliations:** 1 Department of Epidemiology and Public Health, University College Cork, Western Gateway Building, Western Rd, Cork, Ireland; 2 Department of Surgery, Mubarak Al-Kabeer University Hospital, Kuwait, Kuwait; 3 Nuffield Department of Population Health, University of Oxford, Rosemary Rue Building, Old Road Campus, Headington, Oxford OX3 7LF, UK

**Keywords:** Quality of life, Patient-reported outcomes, Hip replacement, Knee replacement, Oxford hip score, Oxford knee score

## Abstract

**Background:**

It is unclear if there is a clinically important improvement in the six to 12-month recovery period after hip and knee replacement. This is an obvious gap in the evidence required by patients undergoing these procedures. It is also an issue for the English PROMs (Patient-Reported Outcome Measures) Programme which uses 6-month outcome data to compare the results of hospitals that perform hip and knee replacements.

**Methods:**

A systematic review of studies reporting the Oxford Hip Score (OHS) or Oxford Knee Score (OKS) at 12 months after surgery was performed. This was compared with six-month outcome data collected for 60, 160 patients within the English PROMs programme. A minimally important difference of one standard error of the measurement, equivalent to 2.7 for the OHS and 2.1 for the OKS, was adopted.

**Results and discussion:**

Six studies reported OHS data for 10 different groups containing 8,308 patients in total. In eight groups the change scores reported were at least 2.7 points higher than the six-month change observed in the PROMs programme (20.2 points). Nine studies reported OKS data for 13 different groups containing 4,369 patients in total. In eight groups the change scores reported were at least 2.1 points higher than the six-month change observed in the PROMs programme (15.0 points).

**Conclusions:**

There is some evidence from this systematic review that clinically important improvement in the Oxford hip and knee scores occurs in the six to 12 month recovery period. This trend is more apparent for hip than knee replacement. Therefore we recommend that the English Department of Health study the impact on hospital comparisons of using 12- rather than six-month outcome data.

## Background

Hip and knee replacement are common surgical procedures used in the treatment of severe osteoarthritis. The Oxford Hip Score [1] (OHS) and Oxford Knee Score [2] (OKS) have been extensively used in the last twenty years to assess the patient-reported outcome of these operations. Both measures are reliable, valid and responsive to change [1,2]. Each contains 12 questions about joint pain and function in the past four weeks which are scored from zero to four and summed to produce a total score ranging from 0 to 48 [3]. Higher scores represent better health-related quality of life.

Since April 2009 the Department of Health in England has required the routine collection of the OHS and OKS for patients undergoing hip and knee replacement [4]. This is known as the ‘PROMs’ or Patient-Reported Outcome Measures programme and its main objective is to audit the performance of public and independent surgical providers. A dataset covering nearly 100,000 procedures performed at more than 270 centres is now freely available on the internet. This provides a precise and generalisable estimate of the mean change in the OHS and OKS from before joint replacement to six months after surgery.

The data collection methods for the PROMs programme were derived from a multi-centre pilot study performed in 2007 [5] which recommended the use of outcome assessment at six months after joint replacement. The choice of a six month interval represented a judgement about the earliest time point in the post-operative recovery process at which the average patient has achieved all the clinically important benefits of surgery. As the main purpose of the PROMs programme is the comparison of the performance of different surgical providers it was deemed necessary to measure outcome as early as practicable because of the need to detect deviant performance on a timely basis. The recommendation to measure outcome at six months was based upon clinical consensus rather than evidence about the normal pattern of postoperative recovery and it is possible that too early a time point was chosen for the fair comparison of surgical providers. To date only one small, single centre, longitudinal study has been published that quantifies the magnitude of improvement in the English version of the OHS from six months onwards [6]. This was a randomised controlled trial (RCT) of two hip prostheses in 43 patients and found no important change in the OHS from six to 12 months for either patient group, but an improvement from 12 to 24 months. No such studies have been published for the OKS. In contrast, many studies have reported the level of improvement in the OHS and OKS from before surgery to 12 months after surgery. Some of these studies have also measured improvement at later time points. A systematic review of these studies would allow for a generalisable estimate of the amount of change in the OHS and OKS that is experienced by 12 months and onwards after hip and knee replacement and whether this is significantly larger from a clinical perspective than that measured at six months by the PROMs programme. Of worldwide benefit to patients, clinicians and policy makers would be a synthesis of evidence about the natural trajectory of recovery in the first year after surgery. This would give patients the information they need to plan their return to normal activities and clinicians and policy makers the information they need to properly design a comparative audit such as the English PROMs programme. At present it is striking that there is little evidence-based information available about the point at which all clinically important improvement after surgery has ended. This paper combines a systematic review of the available literature with data extracted from the English PROMs programme dataset to test the hypothesis that there is clinically important improvement in the six to 12-month recovery period in patient-reported outcomes after hip and knee replacement.

## Methods

All prospective longitudinal studies which reported change in the OHS or OKS from before to after surgery were screened for review. Studies which included adults with osteoarthritis of the hip or knee undergoing total hip or knee replacement were included regardless of the type of prosthesis, or rehabilitation protocol used. Studies which focused on hip resurfacing or unicompartmental knee replacement procedures were not included in the review. Only longitudinal studies that reported the mean OHS or OKS before and at 12 months after surgery were included. To reduce the amount of heterogeneity in the review, studies were excluded if the sample included patients undergoing revision procedures as this is a less effective procedure than primary surgery [7]. We also excluded studies that used non-English versions of the OHS and OKS as this was considered a further source of heterogeneity. All potentially eligible studies were examined for the possibility of multiple-publication of data on a single patient sample. Where such studies were discovered only the original publication was included.

MEDLINE and the Web of Knowledge were searched for studies published in the period up to and including June 2011. In MEDLINE we reviewed all studies retrieved by searching for the text terms “Oxford hip”, “Oxford knee” and “Oxford 12” in all fields. No time, language or study design restrictions were used. In the Web of Knowledge we searched for all studies which cited the original validation papers [1,2] of the OHS and OKS. We used a conservative approach to title and abstract review and discarded only those studies which obviously did not satisfy our inclusion and exclusion criteria.

The PubMed search revealed a total of 94 citations that included the term “Oxford hip” and 129 that included the term “Oxford knee”. The Web of Knowledge search identified 202 articles which had cited the original validation paper for the Oxford Hip Score and 199 articles which had cited the original paper for the Oxford Knee Score. Citations were managed using the Endnote software package. Following the elimination of duplicate papers, independent screening of the abstracts for these citations by two reviewers (JB and HB) identified 35 possibly relevant papers for the Oxford Hip Score and 31 possibly relevant papers for the Oxford Knee Score. Full text copies of these papers were obtained and independently reviewed for relevance by JB and HB. Papers which covered the wrong patient population or surgical procedures, presented insufficient data, were literature reviews or covered the same sample of patients as another paper were eliminated at this stage. Following discussion it was agreed that six papers for the Oxford Hip Score and nine papers for the Oxford Knee Score should be included in the final systematic review.

Two reviewers (HB and JB) independently extracted data on the OHS and OKS at baseline, 12-months and any subsequent time points from each included study. Any differences in the data extracted were resolved through discussion. For many studies it was necessary to convert the reported means from the original 12-60 scale (60=most severe) to the new 0-48 scale (0=most severe) which is now the accepted standard [3]. The formula for converting scores was y = 60 - x where y is the desired score on the new scale and x is the score from the original scale. Change scores were extracted if reported and are of the same magnitude, irrespective of whether the 12-60 or 0-48 scoring system is used. If the change score was not reported it was derived by a simple subtraction of the reported pre-operative mean from the reported post-operative mean. Results for multiple patient groups are presented for comparative studies unless scores for all patient groups were presented in aggregate form somewhere in the paper.

The English PROMs Programme uses two important methods to minimise bias. First, all eligible patients are invited to take part in the Programme so that local hospital staff do not introduce selection bias. Second, all questionnaires are completed by the patient on their own so that interviewer bias on the part of local hospital staff or clinicians is avoided. To assess the extent to which the studies iin our systematic review had the same level of methodological quality as the English PROMs Programme, two authors (HB and JB) independently reviewed the methods used in the selection of study participants (to assess for potential selection bias) and the methods used in the administration of the OHS and OKS (to assess for potential interviewer bias).

To enable a comparison with data from the PROMs programme the Health and Social Care Information Centre in England was asked to provide the mean six-month improvement in the OHS and OKS for patients undergoing primary surgery. This request was necessary because although information about the PROMs programme is freely available on the internet it does not distinguish between patients undergoing primary and revision surgery. The data extract captured the primary part of the relevant Operating Procedure Codes (OPCS version 4.3) for total primary hip and knee replacement procedures and therefore matched the patient population covered by our systematic review.

The primary objective of this systematic review was to compare the post-operative improvements in the OHS and OKS observed at six months by the PROMs programme with the improvements at 12 months reported in the published literature. A direct statistical comparison was not possible because there was insufficient information in the published literature to derive a pooled estimate of change with confidence intervals at 12 months using a meta-analysis. Instead we provide a narrative synthesis which compares the clinical importance of change at six and 12 months. When comparing mean change scores we defined a minimally important difference (MID) as equal to or greater than one Standard Error of the Measurement (SEM). The SEM was chosen because it is relatively constant when measured in different samples of patients and therefore lends itself to comparison of results from different studies. The SEM has been estimated as 2.7 points for the OHS and 2.1 for the OKS [5]. A second objective of the review was to compare the difference between 12-month outcome scores and outcome scores recorded at later time points.

## Results

A mean improvement score of 20.2 points (95% CI: 20.1-20.3) for the OHS and 15.0 points (95% CI: 14.9-15.1) for the OKS was reported by the Health and Social Care Information Centre based on data extracted in July 2012 from the Hospital Episode Statistics dataset for the English PROMs programme. The mean pre-operative scores were 18.2 (95% CI: 18.1-18.3) for hip replacement patients and 19.0 (95% CI: 18.9-19.1) for knee replacement patients. This information relates to 29,544 patients undergoing primary hip replacement surgery and 30,616 patients undergoing primary knee replacement surgery between April 2010 and May 2011. Thus any 12-month mean change scores reported in the literature as greater than 22.9 (i.e. 20.2 plus a MID of 2.7) for the OHS and 17.1 (i.e. 15.0 plus a MID of 2.1) for the OKS were taken as evidence that important improvement in these scores may occur after a six month outcome assessment.

Six published studies [6,8-12] reported the change in mean OHS from before to 12 months after primary hip replacement for 10 different groups containing 8,308 patients in total (Table 1). Three studies were RCTs, three were prospective cohort studies and all were conducted in the UK. Surgery was described as total hip replacement (THR) in seven patient groups and total hip arthroplasty (THA) in three patient groups. The mean improvement in the OHS was greater than 20.2 in all 10 patient groups and greater than 22.9 in eight patient groups. The patients covered by the published studies tended on average to have a lower OHS before surgery than patients in the English PROMs study. Nine of the 10 patient groups reported in the literature had lower baseline OHS scores than the English PROMs patients and this difference was clinically important in seven of the 10 groups (see Table 1).

**Table 1:** Studies that provide Oxford Hip change scores at 12 months for patients undergoing hip replacement

**Study reference**	**Location**	**Description of surgery**	**Study design**	**Number of patients**	**Baseline OHS**	**12-month OHS**	**OHS change**
Hajat (2002) [8]	England	THR	Prospective cohort	6572	15.3	38.1	22.8
Palan (2009) [9]	England	THR by trainer	Prospective cohort	795	16.6	39.9	23.3
Palan (2009) [9]	England	THR by trainee	Prospective cohort	449	15.2	38.5	23.3
O’Brien (2009) [10]	Northern Ireland	THA	Prospective cohort	335	10.8	38.9	28.1
Simpson (2010) [6]	England	THR: Furlong HAC stem	RCT	23	13.2	40.1	26.9
Simpson (2010) [6]	England	THR: Furlong Active Femoral stem	RCT	20	13.1	43.4	30.3
Thomas (2011) [11]	England	THR: high cross linked polyethylene	RCT	22	12.0	46.7	34.7
Thomas (2011) [11]	England	THR: ultra-high molecular weight	RCT	22	10.0	47.1	37.1
Desai (2011) [12]	England	THA: leg-length discrepancy technique	RCT	35	17.6	41.9	24.3
Desai (2011) [12]	England	THA: standard technique	RCT	35	18.8	39.7	20.9

Three of the above studies also collected OHS data at time points after 12 months. All were RCTs and scores were reported for six different patient groups. Only one of the six patient groups showed a further minimally important improvement on the OHS after 12 months. The first study collected OHS data annually for five years after surgery for patients operated on by either a trainee or a trainer [9]. The mean improvements from baseline in the OHS reported in successive post-operative years for the trainer-operated patients were 23.3, 23.1, 23.4, 23.9 and 23.9 respectively. The equivalent scores for the trainee-operated group were 23.3, 22.5, 23.3, 23.5 and 24.0. The second study reported the mean improvement from baseline in the OHS at 12 and 24 months after surgery for patients receiving two different versions of a cementless femoral stem [6]. The mean OHS change for the first group was 26.9 at 12 months and 31.4 at 24 months, a minimally important improvement during the second post-operative year. The statistical significance of this improvement is not reported. The mean OHS improvement for the second group was 30.3 at 12 months and 31.6 at 24 months. The third study reported OHS data at 12 months and seven years after surgery for patients receiving two different versions of acetabular liner [11]. A minimally important decline in the OHS was observed from 12 months to 7 years in both groups. The mean decline was 5.3 points in the first group and 6.0 points in the second group. The statistical significance of this decline is not reported.

Nine published studies [10,13-20] reported the change in mean OKS from baseline to 12 months after primary knee replacement surgery for 13 different groups containing 4,369 patients in total (Table 2). Three studies were RCTs and six were prospective cohort studies. One study was carried out in Australia and the rest were conducted in the UK. Surgery was described as total knee replacement (TKR) in two patient groups and total knee arthroplasty (TKA) in 11 patient groups. The mean improvement in the OKS was greater than 15.0 in eleven patient groups and greater than 17.1 in eight patient groups. In general, the mean pre-operative OKS of the patient groups covered by the published literature was similar to that seen in the English PROMs programme (see Table 2).

**Table 2:** Studies that provide Oxford Knee change scores at 12 months for patients undergoing knee replacement

**Study reference**	**Location**	**Description of surgery**	**Study design**	**Number of patients**	**Baseline OKS**	**12-month OKS**	**OKS change**
Kamat (2008) [13]	England	TKA: computer assisted	Prospective cohort	263	19.6	35.6	16.0
Kamat (2008) [13]	England	TKA: standard instrumentation	Prospective cohort	302	20.1	34.2	14.1
Unitt (2008) [14]	England	TKR	Prospective cohort	394	17.3	36.1	18.8
KAT Group (2009) [15]	UK	TKA	RCT	2352	18.0	34.2	16.2
O’Brien (2009) [10]	Northern Ireland	TKA	Prospective cohort	253	12.9	33.9	21.0
Smith (2010) [16]	Scotland	TKA: computer navigated by novice	Prospective Cohort	50	18.0	37.0	19.0
Smith (2010) [16]	Scotland	TKA: computer navigated by expert	Prospective Cohort	50	17.0	40.0	23.0
Hanusch (2010) [17]	England	TKA: fixed-bearing	RCT	55	19.6	38.6	19.0
Hanusch (2010) [17]	England	TKA: rotating-platform	RCT	50	19.8	39.0	19.2
Reddy (2010) [18]	England	TKA	Prospective cohort	379	18.9	39.0	20.1
Hossein (2011) [19]	England	TKA: Press Fit Condylar prosthesis	RCT	40	18.3	30.5	12.2
Hossein (2011) [19]	England	TKA: Medial Rotation prosthesis	RCT	40	18.4	34.1	15.7
Naylor (2011) [20]	Australia	TKR	Prospective cohort	141	21.1	39.3	18.2

Three of the eligible studies collected OKS data at time points after 12 months. Two were RCTs, one was a prospective cohort study and scores were reported for five different patient groups. Only one of the five patient groups showed a minimally important improvement on the OKS after 12 months. The first study collected OKS data annually after surgery for patients operated on using standard and computer assisted techniques [13]. The mean improvements in the OKS from baseline in successive post-operative years for the standard-technique patients were 14.1, 15.0, 15.4, 15.6 and 14.7 respectively. The equivalent scores for the computer-assisted group were 16.0, 15.9, 14.7, 16.2 and 13.5. This indicates a minimally important decline in the OKS from four to five years for the computer-assisted group. The statistical significance of this change is not reported in the paper and it should be noted that there was substantial attrition in follow-up in this study and the five-year data relate to only 15 patients in the standard-technique group and 13 patients in the computer-assisted group. The second study provided OKS data at 12 months and two years after surgery for patients receiving various types of knee replacement [15]. The change from baseline in the mean OKS was 16.2 points at 12 months and 16.8 points at two years indicating no minimally important improvement during the second post-operative year. The third study reported OKS data at 12 months and two years after surgery for patients receiving two different types of ball and socket design [19]. The mean OKS was 12.2 points higher after 12 months and 12.6 points higher after two years for the first group. The equivalent scores for the second group were 15.7 and 15.4 respectively. This indicates that no minimally important improvement occurred from 12 to 24 months after surgery in both groups.

The risk of selection bias in the recruitment of study participants was considered low in four of the hip replacement studies [6,8,11,12] and eight of the knee replacement studies [13-20] as there was clear evidence that consecutive eligible patients were invited to take part. In two of the hip replacement studies [9,10] and one of the knee replacement studies [10] it was unclear whether this was true. The risk of interviewer bias was considered low in five of the hip replacement studies [6,8-11] and six of the knee replacement studies as there was clear evidence that patients completed their questionnaires without the risk of interference from a member of the research team or local hospital staff. In one of the hip replacement studies [12] and three of the knee replacement studies [14,17,19] it was unclear if this was true.

## Discussion

This systematic review has shown that most of the improvement in the OHS occurs in the first six months after hip replacement surgery. However, the published literature provides fairly consistent evidence (from eight out of 10 patient groups) of a minimally important difference between the benefits of surgery experienced at six and 12 months. The published evidence for knee replacement surgery presents a less consistent message. While it is clear that most of the improvement in the OKS occurs in the first six months after knee replacement surgery there is inconsistent evidence (from eight out of 13 patient groups) of a minimally important difference occurring between the benefits measured at six and at 12 months. In both hip and knee replacement the limited available evidence suggests that no difference exists between outcomes assessed at 12 months compared with later time points. This is the first systematic review to synthesise evidence about the natural trajectory of recovery after hip and knee replacement. The findings of the study should be used to provide information to patients about the length of time it takes the average patient to accrue the clinically important benefits of surgery.

The interpretation of change in the OHS and OKS requires an understanding of what has been defined as a MID in this study. There is no consensus about the most appropriate method of estimating change values that are considered to be of minimal importance for the OHS and OKS and it should be noted that if a different method had been applied the interpretation of the evidence in this review would change. For example, MID values of 8.4 for the OHS and 3.8 for the OKS have been produced using an ‘anchor-based’ technique [21]. If these values had been used only three out of 10 hip replacement groups and six out of 13 knee replacement groups would have change scores at 12 months that could be considered different from those seen in the English PROMs data at six months after surgery. The lower MID values used in this review were chosen in part because, given the high stakes involved in comparing and publically reporting on the performance of healthcare providers, we consider the danger of missing a real difference between six and 12-month change scores to outweigh the consequences of a ‘false alarm’.

It is important to bear in mind that this review only captures the improvements in pain and function that are measured by the OHS and OKS. As the mean post-operative OHS and OKS reported in the literature at 12 months after surgery is in many cases close to the maximum possible score for these measures, particularly in the case of the OHS, it is possible that ceiling effects mask the true level of improvement between six and 12 months.

It must also be stressed that the literature on the English language version of the OHS and OKS is almost exclusive to the United Kingdom and the generalisability of this review must be considered in that light. There is little evidence available to suggest marked differences in the outcomes achieved by hip and knee replacement patients in different countries but one paper [22] published in 2004 has reported that knee replacement patients in the United Kingdom have significantly worse functional outcomes but similar pain relief at both the one and two year follow-up points compared with those from the United States and Australia. The one non-UK study [20] reported in our review found that the mean change in OKS for a sample of Australian patients undergoing knee replacement was 18.2 points which is comfortably within the range of 12.2 to 23.0 for the 12 UK patient groups. This provides some support for including the Australian study in our review.

A weakness of the review is the difficulty in making inferences about longitudinal trends from cross-sectional evidence. It is not possible to conclude that the outcomes reported at 12 months in the reviewed studies are an accurate representation of the outcomes that would be reported by patients in the English PROMs programme were they to have a 12 month outcome assessment. It is possible, for example, that the reviewed studies represent a subset of patients more likely than the average patient to experience improvement after surgery. Table 1 demonstrates that the hip replacement patients covered by this literature review tended to have a lower pre-operative OHS than the patients reported by the English PROMs programme. Patients with lower pre-operative scores have greater room for improvement from a purely statistical perspective [21], and this may explain the differences between six and 12 month OHS data we have observed. It is of note that the pre-operative OKS data reported in the literature is similar in general to that reported by the English PROMs programme (Table 2). This may explain why the evidence for a difference between six and 12 month improvement is much weaker in knee replacement than in hip replacement. A further weakness of the review is the heterogeneity of patient groups and study designs presented. This, as well as the absence of published data about the precision of change scores, prohibited a meta-analysis and limited the review to a narrative synthesis. Finally, although the quality of the reviewed studies is generally high with little evidence of selection or interviewer bias, a small number of studies did not attain the same methodological standards as are evident in the English PROMs programme. Notwithstanding these criticisms the evidence presented is the best available guide to improvements in the OHS and OKS after primary hip and knee replacement in the absence of a generalisable study which measures outcome at both six and 12 months after surgery.

A somewhat surprising outcome of this review is the apparent difference in the trajectory of recovery experienced by hip and knee replacement patients. Contrary to conventional opinion the published evidence suggests that there may be less improvement after six months in patients undergoing knee replacement surgery than in those undergoing hip replacement. This may be a reflection of the content of the OKS rather than any real difference in the recovery speed of the two patient groups. For example, it is possible that the functional improvements that are most delayed after knee replacement are not represented in the OKS. It is also possible that patients undergoing knee replacement have other clinical conditions such as bilateral pain and obesity that limit their prospect of reaching the plateau of function achieved by patients undergoing hip replacement [23].

The primary purpose of the English PROMs programme is the detection of deviant performance by surgical providers. Implicit in the methods is the assumption that the performance of these providers can be fairly judged at six months after surgery as all the clinically important benefits of surgery have accrued. If this is not the case it is possible that some providers are being unfairly assessed. This would be possible if, for example, the patients treated at a particular unit ultimately achieve an acceptable or superior outcome compared to patients treated at other units but they do so at a slower pace. This could happen because, for example, the unit in question employs surgical techniques or rehabilitation protocols that trade the speed of recovery for the quality of ultimate outcome.

## Conclusions

There is some evidence from this systematic review that clinically important improvement in the Oxford hip and knee scores occurs in the six to 12 month recovery period. This trend is more apparent for hip than knee replacement. We recommend therefore that the Department of Health in England study the impact on provider comparisons of using both six and 12-month outcome data. This could be achieved by collecting data at both time points for a limited time period. The objective of such a study would be to derive a transition matrix for the interaction of follow-up time and deviant performance. This would quantify the probability of a provider making the transition from one performance category to another based on six versus 12-month outcome data.

## Competing interests

The authors declare that they have no competing interests.

## Authors’ contributions

JB conceived the study, supervised the systematic review, led the extraction of data from the English PROMs Programme and drafted the manuscript. HB performed the systematic review. JD assisted with the systematic review and provided methodological insights regarding interpretation and analysis of the Oxford scores. All authors read and approved the final manuscript.

## Authors’ information

John Browne was the Principal Investigator on the methodological work that developed the English PROMs programme for elective surgery. Jill Dawson was the Co-developer of the Oxford Hip and Knee scores. Hamad Bastaki is a junior doctor with an interest in the use of patient-reported outcome measures in clinical audit.
